# Development of Insert Condition Classification System for CNC Lathes Using Power Spectral Density Distribution of Accelerometer Vibration Signals

**DOI:** 10.3390/s20205907

**Published:** 2020-10-19

**Authors:** Yi-Wen Huang, Syh-Shiuh Yeh

**Affiliations:** 1 Institute of Mechatronic Engineering, National Taipei University of Technology, Taipei 10608, Taiwan; t105408012@ntut.org.tw; 2 Department of Mechanical Engineering, National Taipei University of Technology, Taipei 10608, Taiwan

**Keywords:** insert conditions, accelerometer, power spectral density, CNC lathes

## Abstract

Insert conditions significantly influence the product quality and manufacturing efficiency of lathe machining. This study used the power spectral density distribution of the vibration signals of a lathe machining accelerometer to design an insert condition classification system applicable to different machining conditions. For four common lathe machining insert conditions (i.e., built-up edge, flank wear, normal, and fracture), herein, the insert condition classification system was established with two stages—insert condition modeling and machining model fusion. In the insert condition modeling stage, the magnitude features of the segmented frequencies were captured according to the power spectral density distributions of the accelerometer vibration signals. Principal component analysis and backpropagation neural networks were used to develop insert condition models for different machining conditions. In the machining model fusion stage, a backpropagation neural network was employed to establish the weight function between the machining conditions and insert condition models. Subsequently, the insert conditions were classified based on the calculated weight values of all the insert condition models. Cutting tests were performed on a computer numerical control (CNC) lathe and utilized to validate the feasibility of the designed insert condition classification system. The results of the cutting tests showed that the designed system could perform insert condition classification under different machining conditions, with a classification rate exceeding 80%. Using a triaxial accelerometer, the designed insert condition classification system could perform identification and classification online for four common insert conditions under different machining conditions, ensuring that CNC lathes could further improve manufacturing quality and efficiency in practice.

## 1. Introduction

In machining processes, machine tools operators frequently determine the operating conditions of the cutting tools based on their professional experience. However, this approach cannot increase manufacturing efficiency and is expected to result in unreliable machining quality and long machining times. Therefore, in recent years, developing reliable tool condition monitoring systems has become an important issue in product manufacturing. Tool condition monitoring systems can identify the operating conditions of cutting tools and perform classification correctly so that defective cutting tools can be changed at the appropriate time, enhancing the machining quality and manufacturing efficiency. In this study, an insert condition classification system applicable to lathe machining processes was developed to classify four common insert conditions: built-up edge, flank wear, normal, and fracture. 

The main implementation procedure of tool condition monitoring comprises the selection and mounting of a sensor, signal acquisition and processing, feature capture, and condition classification [1,2], and it can be classified into direct and indirect monitoring. Direct monitoring technologies use measuring instruments, such as three-dimensional surface profilers, optical microscopes, scanning electron microscopes, and charge-coupled device cameras, to inspect the cutting tool conditions directly [3,4]. Direct monitoring technologies have a higher judgment accuracy for tool condition classification than do the indirect types and are sometimes used for offline tool condition inspection. Specifically, when employing these technologies, the cutting tools are extracted from the manufacturing machine and placed on the measuring instruments to inspect the cutting tool conditions directly. Indirect monitoring technologies measure sensing signals corresponding to the insert condition during the machining process, including information, such as cutting force [5,6,7,8,9], vibration [10,11,12], acoustic emissions [13,14,15], temperature [16], and sound [17], and subsequently analyze the signals for tool condition classification. Indirect monitoring technologies have lower tool condition classification accuracy than do direct types; however, they are applicable for online tool condition inspection. Specifically, indirect monitoring technologies can identify and classify the condition of the cutting tools without extracting them from the manufacturing machine. In practical applications, product manufacturers initially use indirect monitoring technologies to identify and classify the conditions of cutting tools online and subsequently use direct monitoring technologies for further inspection. This process can improve the identification of operators and reduce the frequency and time required to change cutting tools, thereby contributing to enhancing the machining quality and manufacturing efficiency. 

A tool condition monitoring system mainly captures sensing signal features and performs tool condition classification. In the sensing signal capture stage, the system performs signal processing of the acquired original sensing signals and subsequently captures the appropriate sensing features from the processed signals. Signal processing and feature capturing are typically performed using time-domain analysis, frequency-domain analysis, time-frequency analysis, and statistical property methods. In the tool condition classification stage, common classification technologies are utilized, such as fuzzy logic [8,18], artificial neural networks (ANNs) [18,19], and support vector machines (SVMs) [20,21].

Kaya et al. used an SVM to develop a tool condition monitoring system that could acquire cutting force, cutting torque, vibration, and acoustic emission signals. This system uses the sensor fusion method to capture time-domain statistical features from the sensing signals and subsequently employs a genetic algorithm to determine the main features of the cutting tool conditions [22]. Wang et al. used a v-SVM to design a tool condition monitoring system that could acquire vibration signals during the cutting process. This system utilizes the locality preserving projection algorithm to reduce the feature dimensions and applies the nearest neighbor rule to select the training samples for modeling [23]. Downey et al. proposed a multiple sensor automatic data acquisition system to acquire vibration, cutting force, and acoustic emission signals, and the system is applicable to the computer numerical control (CNC) turning center of a real-time production environment. To ensure the quality of acquired signals, a cutting force sensor is mounted on the lower part of the tool holder of a tool condition monitoring system, and an accelerometer and acoustic emission sensors are mounted on its upper part [24]. González-Laguna et al. analyzed vibration signals and found that their root mean square and the amplitude of a fast Fourier transform were correlated to the condition of the cutting tools in steel turning operations. Subsequently, they used the results to design a tool wear condition monitoring system [25]. Arslan et al. studied the relationships among the statistical properties of vibration signals and found that the tool wear condition and workpiece surface roughness could be estimated using the crest factor, root mean square, and Kurtosis value of the vibration signals [26]. Salimiasl and Özdemir evaluated the performance of tool condition monitoring systems using the three most well-known classification methods: ANNs, fuzzy logic, and the least square method, and concluded that the tool condition model built using ANNs was the most accurate [18]. Caggiano acquired the cutting force, acoustic emission, and vibration signals during turning processes and captured statistical features from these sensing signals to build an ANN-based tool wear model. This model uses principal component analysis (PCA) to reduce feature dimensions and can estimate tool flank wear during turning processes accurately [27]. 

Presently, tool condition monitoring systems universally employ cutting force, vibration, and acoustic emission signals. However, dynamometers used for accurate measurement of cutting force and cutting torque tend to be expensive and large; they occupy part of the manufacturing machine’s working space and influence the movements of the cutting tools. If a force sensor is mounted on the tool holder of a tool condition monitoring system, the structural rigidity is expected to be insufficient, and cutting path interference can occur. Kulandaivelu et al. found that acoustic emission signals were related to the flank wear advance along the side edge of an insert; therefore, an acoustic emission sensor should be installed on the side face of a tool holder [28]. However, in this case, there may be cutting path interference, leading to a collision. Consequently, in this study, accelerometer vibration signals applicable to the machining processes of CNC lathes were measured. In addition, based on the excellent signal feature reduction capability of PCA and the outstanding modeling and classification performance of ANNs, in this study, PCA was used to reduce the feature dimensions of the accelerometer vibration signals, and ANNs were employed for modeling and classification. 

In this study, the power spectral density (PSD) was utilized to analyze the accelerometer vibration signals. It was found that different insert conditions had various feature magnitudes at certain frequencies and that different machining conditions could influence the distribution of the feature magnitudes. To achieve the applicability of the insert condition feature capture and classification method designed in this study to different machining conditions, an L9 orthogonal array was used for the lathe machining plan to obtain the experimental data for the insert condition modeling. Because an L9 orthogonal array has nine machining conditions, correspondingly, nine insert condition models were built in this study. In the insert condition modeling stage, the PSD distribution magnitude featured numerous accelerometer signals. In this study, PCA was employed to reduce the feature dimensions, following which the principal features were utilized for backpropagation neural network (BPNN) modeling. Because nine insert condition models were built based on the experimental plan of the L9 orthogonal array, a machining model fusion mechanism needed to be designed so that these models could be used for classification under different machining conditions. In this study, a BPNN was employed to design the weight functions between the machining conditions and the insert condition models, which were applicable to the machining model fusion mechanism. Lathe machining was performed to validate the feasibility and efficiency of the proposed approach and design. Based on the lathe machining results, under the machining conditions of the experimental plan and in the range of the cutting force, the correct classification rate of the insert condition classification system designed in this study was higher than 80%. Moreover, this rate was significantly better than that of the Autoencoder+Softmax learning system. The purpose of this study includes the following:

- Designing an insert condition classification system that can be used to identify and classify four common lathe machining insert conditions—built-up edge, flank wear, normal, and fracture.
- Using the magnitude features of the PSD distribution of the signals obtained from a lathe machining accelerometer to identify and classify the insert conditions under different machining conditions.
- Performing cutting tests with different machining conditions on a CNC lathe to evaluate the feasibility and performance of the insert condition classification system developed in this study from different machining aspects.

The rest of this paper is organized as follows. Section 2 describes the experimental equipment used in this study and the lathe machining plan. Section 3 presents the insert condition modeling approach, including the frequency segment selection and the insert condition model building. Section 4 describes the machining model fusion mechanism design approach. Section 5 presents the lathe machining results of the insert condition classification system developed in this study and the comparative experiment results of the Autoencoder+Softmax learning system. In addition, the validation of both the feasibility and the performance of the system designed in this study is discussed. Section 6 concludes this paper. 

## 2. Experimental Setup and Experiment Plan

**Figure 1:**
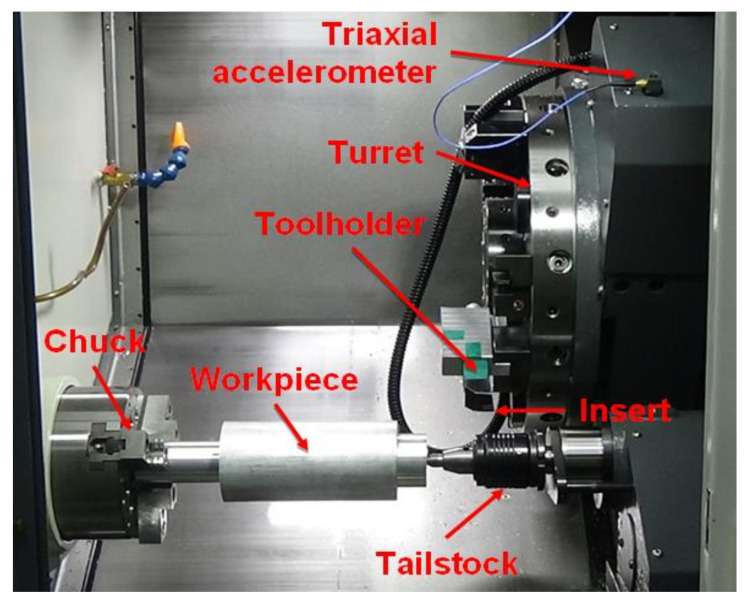
Installation architecture of lathe machining equipment.

**Figure 2:**
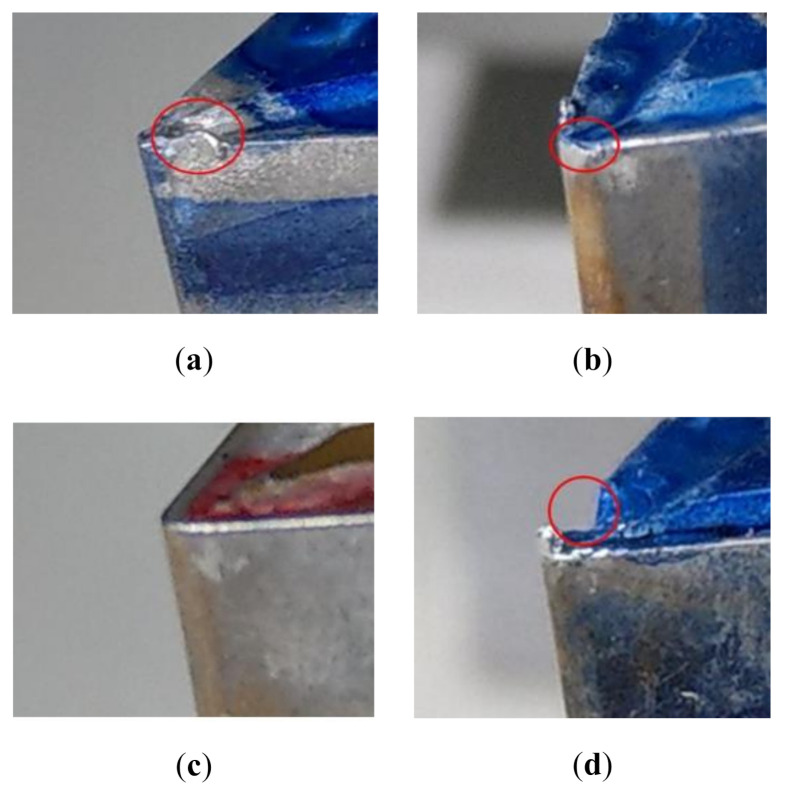
Four insert conditions. (**a**) Built-up edge; (**b**) Flank wear; (**c**) Normal; (**d**) Fracture.

As shown in Figure 1, this study used a CNC lathe for machining. The workpiece was clamped by a 3-jaw chuck and supported by a tailstock. A tool holder with an insert was mounted on a turret, and the movement of the turret was controlled by a CNC controller. During lathe machining, the workpiece rotated, and the turret moved along the workpiece so that the insert could perform the cutting operation. The workpiece with a 90 mm diameter and 300 mm length was made of the commonly used mechanical manufacturing material, Al6061. Inserts with different conditions were installed on the tool holders for lathe machining. The insert was made of tungsten carbide. In this study, inserts with different conditions were collected from several manufacturers of mechanical parts. Figure 2 shows the four insert conditions—built-up edge, flank wear, normal, and fracture. The phenomenon of material building up on the edge of an insert denoted the built-up edge. During lathe machining, the high temperature usually occurred at the interface between the workpiece and the insert. Therefore, the built-up edge insert condition could occur, in which the material on the insert edge breaks away from the insert, and the insert causes a fracture. Due to the erosion in the contact portions between the insert and the workpiece, flank wear could gradually occur at the cutting edge of an insert. Fracture insert condition generally occurs when the cutting force becomes significantly large in lathe machining. The sensitivity of the triaxial accelerometer sensor was 100 mV/g, and it was installed on the machine saddle to measure the accelerometer signals in the machining processes. The sampling frequency of the data acquisition devices was 4096 Hz. A laptop computer performed the frequency-domain analysis of the acquired accelerometer signals, and the obtained frequency-domain features were saved for the subsequent analysis and system design. Each experiment used an infrared non-contact thermometer to measure the insert temperature to ensure the insert temperature variance was lower than ±5 °C in the lathe machining process. Table A1 in Appendix A lists the devices of the experimental setup, and Table A2 in Appendix B lists the composition of the Al6061 used in this study.

As machining and insert conditions significantly influence the variance in accelerometer signals, this study set three levels of machining conditions (i.e., cutting speed, depth of cut, and cutting feed, as listed in Table 1) as control factors. As summarized in Table 2, L9 orthogonal array machining experiments were planned. The L9 orthogonal array was employed to plan the machining experiments, and accordingly, nine experiments were conducted on the four inserts under different conditions. Accelerometer signals were recorded in each machining experiment. 

## 3. Insert Condition Modeling

### 3.1. Frequency Segment Selection

**Figure 3:**
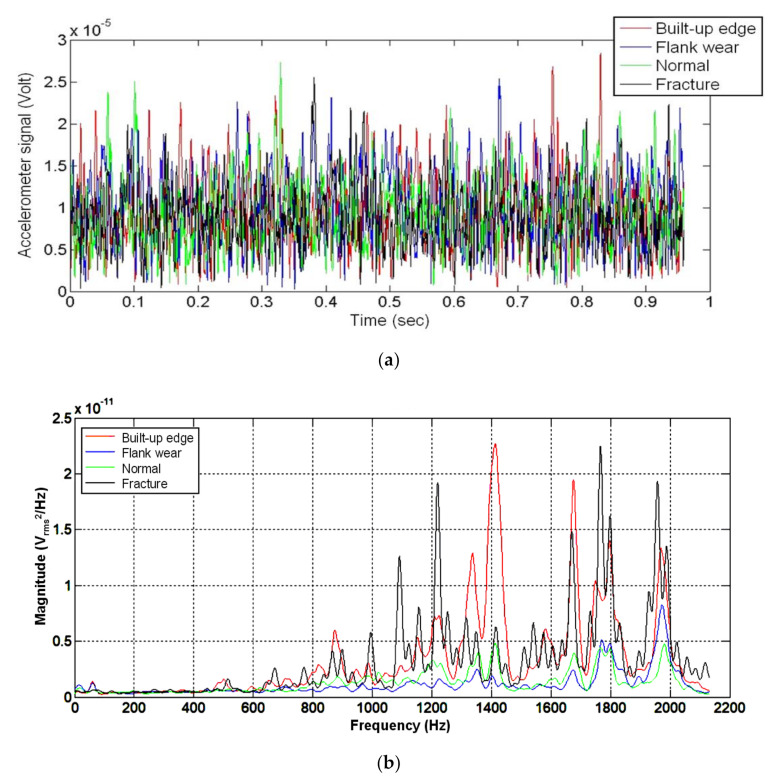
Accelerometer signal time-domain and resultant power spectral density (PSD) distribution diagrams of different insert conditions. (**a**) Time-domain diagram of different insert conditions; (**b**) Resultant PSD distribution diagram of different insert conditions.

The cutting tool conditions could be determined in terms of accelerometer time-domain signals. Figure 3a shows the resultant time-domain signals under the built-up edge, flank wear, normal, and fracture insert conditions. It was challenging to observe differences among the four insert conditions from the time-domain signals. As shown in Figure 3b, this study obtained the resultant frequency-domain PSD distributions of the accelerometer signals and found notable differences between the four insert conditions. The resultant PSD distributions of the accelerometer signals were analyzed based on which the apparent magnitude features of the built-up edge, flank wear, normal, and fracture insert conditions were determined for the subsequent modeling and condition classification.

**Figure 4:**
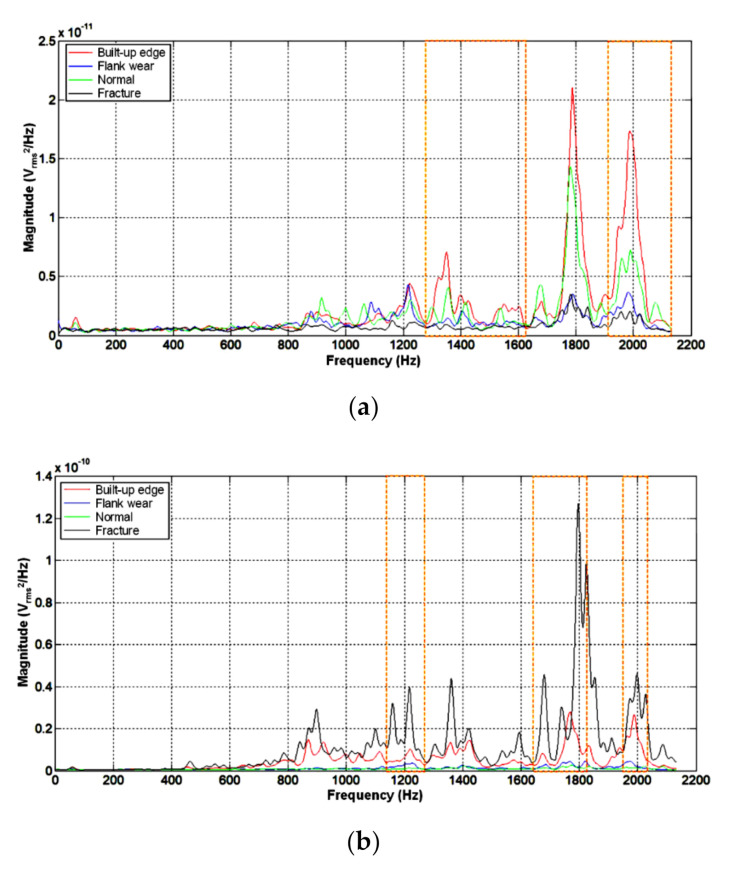
Resultant PSD distribution diagrams of different machining conditions. (**a**) Machining condition 1; (**b**) Machining condition 9.

Because a triaxial accelerometer was used in the study, the resultant PSD distribution of the accelerometer signals could be calculated using the following equation:

(1) $$ACC=\sqrt{ACC_{x}{}^{2}+ACC_{y}{}^{2}+ACC_{z}{}^{2}},$$

where ACC represents the resultant PSD distribution for the subsequent modeling and condition classification. Further, $ACC_{x}$, $ACC_{y}$, and $ACC_{z}$ are the x-, y-, and z-axis PSD distributions of the accelerometer signals, respectively. The resultant PSD distributions of different insert conditions could be significantly influenced by various machining conditions. In this study, the frequency ranges of the different insert conditions were determined with significantly different magnitude features, based on the resultant PSD distributions of the accelerometer signals under different machining conditions. In other words, the frequency ranges of the different insert conditions and those with apparent magnitude differences were selected based on the resultant PSD distributions of the accelerometer signals of the different machining conditions. Furthermore, the resultant PSD magnitudes in these frequency ranges were selected as the features for modeling and condition classification in this study. Figure 4a shows the resultant PSD distribution of the accelerometer signals of the four different inserts performing machining condition 1, as listed in Table 2. The four different inserts presented significantly different magnitude features in the frequency ranges of 1279–1629 Hz and 1916–2132 Hz. Therefore, in this study, 1279–1629 Hz and 1916–2132 Hz were selected as the frequency segments for machining condition 1, and 1.04 Hz was used as the sampling interval to obtain 545 magnitude features. Figure 4b shows the resultant PSD distribution of the accelerometer signals of machining condition 9 obtained utilizing the same approach as above. Based on the resultant PSD distribution of machining condition 9, 389 magnitude features in the frequency ranges of 1139–1269 Hz, 1644–1829 Hz, and 1949–2036 Hz were obtained. The frequency segments were compiled, as shown in Table 3, according to the results of the orthogonal array machining experiments listed in Table 2. Under the different machining conditions, the different insert conditions had varied PSD distribution results. Therefore, the various machining conditions had different numbers and ranges of frequency segments.

### 3.2. Building the Insert Condition Models

As the data volume was large, in this study, PCA was employed to reduce the data dimension [29]. PCA is a method for reducing the dimensions of big data and determining the most significant features. If there is a training set $S=\left\{x_{i},i=1,\cdots ,N\right\}$ and training data $x_{i}\in R^{d}$, the average of the training set, $\bar{X}$, is as expressed in Equation (2).

(2) $$\bar{X}=\frac{1}{N}\sum_{i=1}^{N}x_{i}$$

The covariance matrix of the training set is C, as expressed in Equation (3).

(3) $$C=\frac{1}{N}\sum_{i=1}^{N}\left(x_{i}-\bar{X}\right){\left(x_{i}-\bar{X}\right)}^{T}$$

The eigenvalue, $\lambda_{i}$, and the eigenvector, $v_{i}$, of the covariance matrix, C, are computed and expressed as Equation (4).

(4) $$Cv_{i}=\lambda_{i}v_{i}.$$

The first m eigenvectors, $\left\{v_{i},i=1,\cdots ,m\right\}$, with large eigenvalues $\left\{\lambda_{i},i=1,\cdots ,m\right\}$ are selected to establish the transformation matrix, $W=\left[\begin{array}{cccc} v_{1} & v_{2} & \cdots & v_{m} \end{array}\right]$, where $W\in R^{d\times m}$. The test data, $x\in R^{d}$, are subtracted from the average vector, $\bar{X}$, and the transformation matrix, W, is employed to obtain the transformed data, y, as expressed in Equation (5).

(5) $$y=W^{T}\left(x-\bar{X}\right)=\left[\begin{array}{c} v_{1}{}^{T}\\ v_{2}{}^{T}\\ \vdots \\ v_{m}{}^{T} \end{array}\right]\left(x-\bar{X}\right)=\left[\begin{array}{c} v_{1}{}^{T}\left(x-\bar{X}\right)\\ v_{2}{}^{T}\left(x-\bar{X}\right)\\ \vdots \\ v_{m}{}^{T}\left(x-\bar{X}\right) \end{array}\right]=\left[\begin{array}{c} y_{1}\\ y_{2}\\ \vdots \\ y_{m} \end{array}\right]$$

Therefore, the transformed data, y, can be expressed as the projection of the test data, x, in eigenvectors $\left\{v_{i},i=1,\cdots ,m\right\}$. The projection of eigenvector $v_{1}$ corresponding to the maximum eigenvalue, $\lambda_{1}$, of the test data, x, is called the first principal component, $y_{1}$. Specifically, the transformed data, y, are composed of m principal components $\left\{y_{1}y_{2}\cdots_{,}y_{m}\right\}$. Therefore, PCA can reduce the original dimension of the test data, d, to form transformed data with dimension m, where m≤d. In the application of PCA, the number, m, of the larger eigenvalues (or larger eigenvectors), i.e., the number of principal components, is determined. The common approach calculates the ratio of the total variance, q, to determine the m value, as expressed in Equation (6). The ratio of the total variance, q, needed to be larger than 0.95 to determine the m value, in this study.

(6) $$q=\frac{\lambda_{1}+\lambda_{2}+\cdots +\lambda_{m}}{\lambda_{1}+\lambda_{2}+\cdots +\lambda_{d}}$$

In this study, PCA was employed to obtain several principal components of the magnitude features, and subsequently, ANNs were used to build the insert condition models. A single hidden layer BPNN [30,31] was utilized for the insert condition modeling. The BPNN used the tan-sigmoid and softmax function as the neuron transfer functions and employed the Levenberg–Marquardt algorithm to train the ANNs. Accordingly, the machining experiment plan summarized in Table 2 was completed, and nine insert condition models of the different machining conditions were built in this study.

In the ANN modeling of machining condition 9 specified in Table 2, frequency segments for the accelerometer PSD distribution of the machining experiment results were obtained, as shown in Figure 4b. A total of 389 magnitude features were obtained at this stage. Subsequently, the dimensions of the magnitude features were reduced using PCA, and the principal components were obtained. In this study, the first three principal components (m=3) were selected as the inputs of the BPNN model for machining condition 9. The ratio of the total variance was 0.9621, and the hidden layer used by the BPNN had four neurons. The confusion matrix summarized in Table 4 shows that the correct classification rate of the BPNN model for machining condition 9 built in this study was 100%. The diagonal elements of the confusion matrix reveal that 11 test data were correctly classified. Column 1 of Table 4 shows that three built-up edge test data were correctly classified, and Column 2 of Table 4 indicates that two flank wear test data were correctly classified. Column 3 of Table 4 shows that three test data were correctly classified as normal, and Column 4 of Table 4 indicates that three test data were correctly classified as a fracture. Table 5 lists the BPNN modeling information for the different machining conditions. Clearly, because the machining conditions had a significant effect on the accelerometer PSD distribution, the BPNN models built using these machining conditions were different. In the BPNN models with a single hidden layer, machining condition 6 required more neurons. However, machining condition 8 could build the BPNN model using fewer neurons.

## 4. Machining Model Fusion Mechanism Design

**Figure 5:**
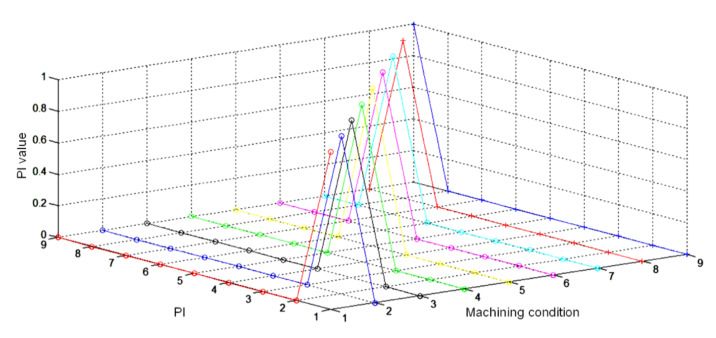
Fusion mechanism established by the backpropagation neural network (BPNN) model.

In this study, nine ANN insert condition models were built according to the orthogonal array summarized in Table 2. To use the insert condition models for the insert condition classification of the different machining conditions, a machining model fusion mechanism was designed. This designed mechanism could establish the weight function between the machining conditions and the insert condition models. In this study, a single hidden layer BPNN was used to establish the machining model fusion mechanism. The fusion mechanism BPNN model had three inputs: cutting speed, depth of cut, and cutting feed, as well as nine outputs, which were the weight values of the nine insert condition models. The hidden layer had six neurons. The training processes of the fusion mechanism BPNN model used the machining conditions listed in Table 2 as the training data. When the input machining conditions of the fusion mechanism BPNN model were the machining conditions of experiment 9 (cutting speed 320 m/min, depth of cut 2 mm, and cutting feed 0.2 mm/rev), the weight value of its output corresponding to the insert condition model of experiment 9 needed to be approximately 1. Moreover, the weight values corresponding to the insert condition models of the other experiments needed to be approximately 0. The relationship between the inputs and outputs of the fusion mechanism BPNN model is shown in Figure 5, where the “machining conditions” correspond to the nine machining conditions listed in Table 2. Furthermore, "PI" and "PI Value" represent the outputs and the weight values of the nine insert condition models, respectively. 

Using the nine insert condition models detailed in Table 5 and the fusion mechanism BPNN model shown in Figure 5, in this study, an insert condition classification system applicable to the different machining conditions, as summarized in Table 6, was designed. The principal components of the PSD distribution of the accelerometer signals were imported into the nine insert condition models to obtain the insert condition classification, $S_{ij}$, as follows:

(7) $${\left.S_{ij}\right|}_{\begin{array}{l} i=1,2,3,4\\ j=1,\ldots ,9 \end{array}}=\left\{0,1\right\}$$

where the value of $S_{ij}$ is 0 or 1. For example, $S_{19}$, $S_{29}$, $S_{39}$, and $S_{49}$ represent the built-up edge, flank wear, normal, and fracture classification results of the accelerometer signals in insert condition model 9 (model 9), respectively, and the value of each classification result is 0 or 1. The machining conditions (cutting speed, depth of cut, and cutting feed) when acquiring the accelerometer signals are imported into the fusion mechanism BPNN model, and the weight values, ${\left.PI_{i}\right|}_{i=1,\ldots ,9}$, of the machining conditions for the nine insert condition models can be obtained. According to the insert condition classification, $S_{ij}$, and the weight values, $PI_{i}$, of the insert condition model, the weighted sum values, $S_{B}$, $S_{W}$, $S_{N}$, and $S_{F}$, for the built-up edge, flank wear, normal, and fracture insert conditions, respectively, can be calculated as follows:

(8) $$S_{B}=\sum_{i=1}^{9}S_{1i}PI_{i},$$

(9) $$S_{W}=\sum_{i=1}^{9}S_{2i}PI_{i},$$

(10) $$S_{N}=\sum_{i=1}^{9}S_{3i}PI_{i},\;\mathrm{and}$$

(11) $$S_{F}=\sum_{i=1}^{9}S_{4i}PI_{i}.$$

The normalized values, $S_{B}\left(\%\right)$, $S_{W}\left(\%\right)$, $S_{N}\left(\%\right)$, and $S_{F}\left(\%\right)$ are as follows:

(12) $$S_{B}\left(\%\right)=\frac{S_{B}}{S_{B}+S_{W}+S_{N}+S_{F}}\times 100\%,$$

(13) $$S_{W}\left(\%\right)=\frac{S_{W}}{S_{B}+S_{W}+S_{N}+S_{F}}\times 100\%,$$

(14) $$S_{N}\left(\%\right)=\frac{S_{N}}{S_{B}+S_{W}+S_{N}+S_{F}}\times 100\%,\;\mathrm{and}$$

(15) $$S_{F}\left(\%\right)=\frac{S_{F}}{S_{B}+S_{W}+S_{N}+S_{F}}\times 100\%.$$

Finally, the insert conditions corresponding to the accelerometer signals can be classified by comparing the normalized values of Equations (12)–(15). 

The execution process and results of the insert condition classification system designed in this study are described below and listed in Table 7. The accelerometer signals were obtained from the machining experiment (with a cutting speed of 318 m/min, depth of cut of 1.8 mm, and a cutting feed of 0.19 mm/rev). First, the accelerometer signals were converted into PSD distributions. Subsequently, they were dealt with in the following ways.

- The frequency segments were executed according to the PSD distributions to obtain the magnitude features.
- The principal components were calculated using PCA according to the obtained magnitude features.
- The insert condition classification, $S_{ij}$, was calculated according to the insert condition models and the principal components.
- The weight values, $PI_{i}$, of the insert condition model were calculated according to the machining model fusion mechanism and the machining conditions.

- The weighted sum values of the built-up edge, flank wear, normal, and fracture insert conditions were calculated using Equations (8)–(11).
- The normalized values of the insert conditions were calculated using Equations (12)–(15).
- According to the normalized values of the insert conditions listed in Table 7, the insert condition corresponding to the accelerometer signals was the built-up edge condition.

## 5. Experimental Results and Discussion

### 5.1. Machining Condition Test Within Range of Machining Experiment Plan

In this study, the machining conditions listed in Table 1 were used for the cutting tests. The machining conditions were a cutting speed of 290 m/min, depth of cut of 1.9 mm, and cutting feed of 0.23 mm/rev. A total of 24 accelerometer signals was obtained from the cutting tests, including six signals each for the built-up edge insert condition, flank wear insert condition, normal insert condition, and the fracture insert condition. These 24 accelerometer signals obtained from the tests were employed for insert condition classification using the classification system designed in this study, and the results are summarized in Table 8.

- There were six signals for the built-up edge insert condition, among which the classification results of five signals were correct, and that of one signal was incorrect. The classification rate was 83.33%.
- There were six signals for the flank wear insert condition, and the classification result was completely correct. The classification rate was 100%.
- There were six signals for the normal insert condition, among which the classification results of four signals were correct, and those of two signals were incorrect. The classification rate was 66.67%.
- There were six signals for the fracture insert condition, among which the classification results of five signals were correct, and that of one signal was incorrect. The classification rate was 83.33%.

The experimental results obtained using a cutting speed of 290 m/min, depth of cut of 1.9 mm, and cutting feed of 0.23 mm/rev showed that the correct classification rate of the insert condition classification system designed in this study was 83.33%. Subsequently, the approaches for the cutting tests under the different machining conditions were the same. The results were as follows:

- The classification rate resulting from the experiment under the machining conditions of a cutting speed of 318 m/min, depth of cut of 1.8 mm, and cutting feed of 0.19 mm/rev was 92.86%.
- The classification rate resulting from the experiment under the machining conditions of a cutting speed of 305 m/min, depth of cut of 1.4 mm, and cutting feed of 0.23 mm/rev was 87.5%.
- The classification rate resulting from the experiment under the machining conditions of a cutting speed of 285 m/min, depth of cut of 1.6 mm, and cutting feed of 0.18 mm/rev was 81.25%.

Therefore, the correct classification rate of the insert condition classification system developed in this study for the machining conditions within the range of the machining experiment plan was at least 80%. 

### 5.2. Machining Condition Test Outside Range of Machining Experiment Plan

To evaluate the insert condition classification rate of the classification system developed in this study for the machining conditions outside the range of the machining experiment plan, a cutting speed of 323 m/min, depth of cut of 2.3 mm, and cutting feed of 0.14 mm/rev were used for the cutting tests. A total of 15 signals were obtained: four signals each for the built-up edge, flank wear, and normal insert conditions, respectively, and three signals for the fracture insert condition. The 15 test signals derived from the experiment were classified by the insert condition classification system developed in this study, and the results are listed in Table 9. The built-up edge, flank wear, normal, and fracture insert condition classification rates were 75%, 50%, 75%, and 0%, respectively. Therefore, the experimental results for the cutting speed of 323 m/min, depth of cut of 2.3 mm, and cutting feed of 0.14 mm/rev showed that the correct classification rate of the classification system developed in this study was only 53.33%. The system built in this study was inapplicable to conditions outside the machining conditions listed in Table 1. 

### 5.3. Cutting Force Experiment

To observe the effect of the cutting force on the classification rate, in this study, cutting tests were performed, wherein the machining conditions for the cutting force experiment were in the range listed in Table 1. Equations (16)–(18) were employed to calculate the cutting force [32,33], wherein F represents the calculated cutting force (N), $k_{c}$ represents the specific cutting force (N/mm^2^), a is the depth of cut (mm), f is the cutting feed (mm/rev), u is a unitless coefficient, $k_{c1}$ represents the specific cutting force (N/mm^2^), $h_{m}$ is the chip thickness (mm), $m_{c}$ is the non-dimensional factor, $\gamma_{0}$ is the rake angle, and $K_{r}$ is the edge angle. For the Al6061 workpiece material and inserts used in this study, $\gamma_{0}$ was −6°, $k_{c1}$ was 700 N/mm^2^, $m_{c}$ was 0.25, $K_{r}$ was 95°, and u was 0.978 [33].

(16) $$F=k_{c}\times a\times f^{u}$$

(17) $$k_{c}=k_{c1}\times h_{m}{}^{-m_{c}}\times \left(1-\frac{\gamma_{0}}{100}\right)$$

(18) $$h_{m}=f\times sin\left(K_{r}\right)$$

In this study, machining with a cutting speed of 306 m/min, depth of cut of 1.1 mm, and cutting feed of 0.22 mm/rev was performed for the cutting tests. The calculated cutting force was 271.4 N. A total of 14 signals were obtained by this experiment: four signals for the built-up edge insert condition, three signals for the flank wear insert condition, three signals for the normal insert condition, and four signals for the fracture insert condition. These obtained 14 test signals were classified using the classification system developed in this study, and the results are listed in Table 10. The built-up edge, flank wear, normal, and fracture insert condition classification rates were 100%, 0%, 67%, and 25%, respectively. The correct classification rate of the experimental results was only 50%. Subsequently, in this study, the same approach as above was employed to test under a cutting speed of 310 m/min, depth of cut of 1.2 mm, and cutting feed of 0.17 mm/rev (a cutting force of 245 N). The classification rate was 42%. The classification rate resulting from the experiment under a cutting speed of 285 m/min, depth of cut of 1.1 mm, and cutting feed of 0.17 mm/rev (a cutting force of 225 N) was 50%. According to the experimental results, the correct classification rate was clearly low. Therefore, the machining conditions were within the range of the machining experiment plan; however, the smaller cutting force reduced the classification rate of the system. 

### 5.4. Comprehensive Discussion

**Figure 6:**
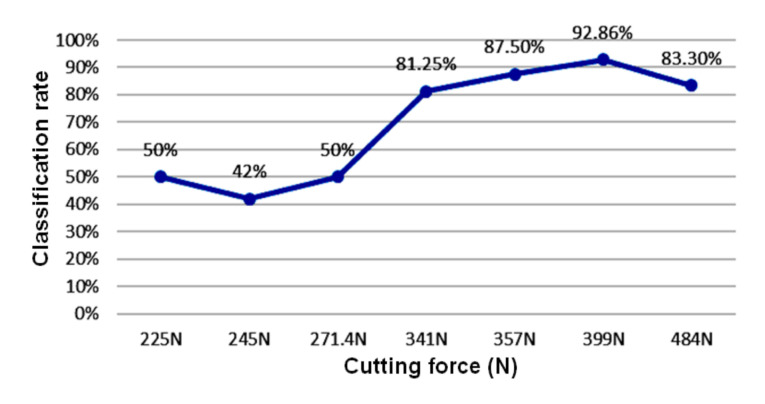
Relationship between cutting force and classification rate.

Summarizing, considering the above-mentioned experimental results, an experimental comparison table was established, which is provided in Table 11. Figure 6 shows the relationship between the cutting force and the classification rate. According to Table 11, the classification rate of the insert condition classification system developed in this study for the machining conditions outside the scope of the experiment listed in Table 1 was only approximately 50%. Specifically, in comparison to the classification rate resulting from the machining conditions within the scope of the experiment, which was higher than 80%, the one resulting from those outside the scope of the experiment was clearly lower. Based on the cutting force test results, although machining conditions within the scope of the experiment were used, the classification rate with a smaller cutting force was lower. As shown in Figure 6, the classification rate was higher than 80% when the cutting force was higher than 341 N, whereas it was only approximately 50% when the cutting force was lower than 271 N. The machining conditions of a cutting speed of 323 m/min, depth of cut of 2.3 mm, and cutting feed of 0.14 mm/rev led to a larger cutting force; however, the classification rate was only 53.3%. This was because the machining conditions were outside the scope of the experiment plan listed in Table 1.

### 5.5. Comparative Experiment Results and Discussion

This study compared two systems for insert condition classification. System 1 was the insert condition classification system developed in this study, and System 2 was the Autoencoder+Softmax learning system [34,35]. The two systems were compared using the same test signals. The autoencoder of the Autoencoder+Softmax learning system was an ANN composed of an input layer, a hidden layer, and an output layer and comprised an encoder network and a decoder network. When training the autoencoder, the encoder and decoder networks were executed to reduce the feature vector dimension of the input signals and reconstruct the signals, respectively. The autoencoder aimed to minimize reconstruction errors. The decoder network was removed after the autoencoder training was completed, and the features captured by the encoder network were imported into the softmax function. The input data of the Autoencoder+Softmax learning system were introduced as the accelerometer PSD distribution (with 2048 magnitude features), and the output was the built-up edge, flank wear, normal, and fracture insert condition classification. There were three hidden layers, the epoch was set as 30, and the batch size was set as 10. The first, second, and third hidden layers used 400, 100, and 10 neurons, respectively. The number of hidden layers, epoch, batch size, and the number of neurons were set by trial and error. Table 12 summarizes the experimental results of the insert condition classification system developed in this study (System 1) and the Autoencoder+Softmax learning system (System 2). 

The experimental results listed in Table 12 indicate that System 2 clearly yielded a lower classification rate than System 1. Based on the cutting tests, when the different machining conditions were used for the experiment, the inserts under varied conditions had different PSD distributions. System 2 could build a model covering all the machining conditions used in Experiments 1–9, as listed in Table 2. However, System 2 was unlikely to correctly classify the insert conditions owing to an insufficient amount of training data. The classification rate of System 2 was lower; however, it was unexpected to be influenced by the cutting force and the machining conditions. In comparison to System 2, System 1 developed in this study was influenced by the cutting force and the machining conditions; however, the correct classification rate was higher than 80% within the scope of the experiment summarized in Table 1. 

## 6. Conclusions

Lathe machining product quality and production efficiency are significantly influenced by insert conditions. This study developed an insert condition classification system by referring to the PSD distribution of accelerometer signals. The system developed in this study used PCA and ANNs, and four common insert conditions—built-up edge, flank wear, normal, and fracture— could be classified under different machining conditions. 

In this study, the PSD distribution of the signals of a lathe machining accelerometer was observed, and it was found that different insert and machining conditions influenced the distributions of the magnitude features of the PSD. To enable the insert condition classification system developed in this study to classify the insert conditions under different machining conditions, an L9 orthogonal array was used to plan the lathe machining experiments. In the insert condition modeling stage, the PSD magnitude feature distributions could determine the frequency range with significantly different magnitude features among the insert conditions. Subsequently, PCA was employed to reduce the feature dimensions, and a BPNN was utilized to build insert condition models. Because the L9 orthogonal array lathe machining experiment plan had nine machining conditions, nine insert condition models were built in this study corresponding to these conditions. For the nine insert condition models, a BPNN was employed to establish the machining model fusion mechanism; thus, the insert condition classification system developed in this study could classify the insert conditions of the different machining conditions. 

The machining model fusion mechanism established the weight function between the machining conditions and the insert condition models. Therefore, the fusion mechanism had three inputs: cutting speed, depth of cut, and cutting feed, as well as nine outputs, which were the weight values of the nine insert condition models. In the machining model fusion stage, the classification system used the BPNN machining model fusion mechanism to calculate the weight values of the nine insert condition models based on the machining conditions when acquiring the accelerometer signals. The insert condition classification system designed in this study could classify the built-up edge, flank wear, normal, and fracture insert conditions according to the classification results of the insert condition models and the weight values of the machining model fusion mechanism. 

The cutting tests performed on a CNC lathe could evaluate the feasibility and the performance of the insert condition classification system developed in this study. The experimental results showed that the correct classification rate of the insert condition was influenced by the machining conditions and the cutting force. The classification rate of the classification system tested for the planned machining conditions exceeded 80%. In addition, the classification system had a higher classification rate when there was a larger cutting force. In comparison to the Autoencoder+Softmax learning system, the classification system designed in this study had a higher classification rate. The experimental results showed that the insert condition classification system developed in this study could be used for lathe machining under different machining conditions and that the correct classification rate of the insert conditions was higher than 80%. The contributions of this study include the following points.

- Development of an insert condition classification system that integrates the insert condition modeling method and machining model fusion mechanism to ensure that the system can be used to identify and classify four common insert conditions online—built-up edge, flank wear, normal, and fracture.
- Development of an insert condition modeling method that considers the resultant PSD distributions of the accelerometer signals, and use of PCA with the frequency segment selection developed in this study to ensure that the data dimension can be significantly reduced in subsequent applications.
- Development of a machining model fusion mechanism that considers both the insert condition models and different machining conditions to ensure that the insert condition classification system developed in this study can be applied to different machining conditions in practice.

This study used an L9 orthogonal array to plan the lathe machining experiments. The correct classification rate of the developed classification system was higher than 80%. However, to further improve the correct classification rate, more insert condition models are required through the use of the orthogonal array with larger dimensions. 

## Figures and Tables

**Table 1:** Machining conditions and levels.

Machining Conditions	Levels		
	1	2	3
Cutting speed (m/min)	280	300	320
Depth of cut (mm)	1	1.5	2
Cutting feed (mm/rev)	0.15	0.2	0.25

**Table 2:** Orthogonal array machining experiment plan.

Experiment no.	Cutting Speed (m/min)	Depth of Cut (mm)	Cutting Feed (mm/rev)
1	280	1	0.15
2	280	1.5	0.2
3	280	2	0.25
4	300	1	0.2
5	300	1.5	0.25
6	300	2	0.15
7	320	1	0.25
8	320	1.5	0.15
9	320	2	0.2

**Table 3:** Frequency segment results of different machining conditions.

Machining Conditions	Frequency Segments	Machining Conditions	Frequency Segments
1	1279 Hz–1629 Hz 1916 Hz–2132 Hz	6	784 Hz–971 Hz 1308 Hz–1442 Hz 1635 Hz–1724 Hz 1914 Hz–2132 Hz
2	1391 Hz–1492 Hz 1905 Hz–2132 Hz	7	1314 Hz–1377 Hz 1644 Hz–1838 Hz
3	1395 Hz–1457 Hz 1613 Hz–1713 Hz 1945 Hz–2132 Hz	8	817 Hz–962 Hz 1174 Hz–1427 Hz 1919 Hz–2132 Hz
4	1279 Hz–1506 Hz 1909 Hz–2132 Hz	9	1139 Hz–1269 Hz 1644 Hz–1829 Hz 1949 Hz–2036 Hz
5	1274 Hz–1469 Hz 1905 Hz–2132 Hz		

**Table 4:** Confusion matrix of the backpropagation neural network (BPNN) model (machining condition 9).

		Predicted Class			
		Built-Up Edge	Flank Wear	Normal	Fracture
Actual class	Built-up edge	3	0	0	0
	Flank wear	0	2	0	0
	Normal	0	0	3	0
	Fracture	0	0	0	3

**Table 5:** Machining conditions and BPNN model information.

Machining Conditions	Number of Neurons	Number of Principal Components	Ratio of Total Variance
1	8	5	0.9960
2	7	3	0.9782
3	4	4	0.9973
4	8	3	0.9935
5	8	4	0.9704
6	12	5	0.9980
7	5	5	0.9629
8	3	2	0.9972
9	4	3	0.9621

**Table 6:** Insert condition classification calculation results.

	Weight Values	Built-Up Edge	Flank Wear	Normal	Fracture
Model 1	$PI_{1}$	$S_{11}$	$S_{21}$	$S_{31}$	$S_{41}$
Model 2	$PI_{2}$	$S_{12}$	$S_{22}$	$S_{32}$	$S_{42}$
Model 3	$PI_{3}$	$S_{13}$	$S_{23}$	$S_{33}$	$S_{43}$
Model 4	$PI_{4}$	$S_{14}$	$S_{24}$	$S_{34}$	$S_{44}$
Model 5	$PI_{5}$	$S_{15}$	$S_{25}$	$S_{35}$	$S_{45}$
Model 6	$PI_{6}$	$S_{16}$	$S_{26}$	$S_{36}$	$S_{46}$
Model 7	$PI_{7}$	$S_{17}$	$S_{27}$	$S_{37}$	$S_{47}$
Model 8	$PI_{8}$	$S_{18}$	$S_{28}$	$S_{38}$	$S_{48}$
Model 9	$PI_{9}$	$S_{19}$	$S_{29}$	$S_{39}$	$S_{49}$
Weighted sum values	―	$S_{B}$	$S_{W}$	$S_{N}$	$S_{F}$
Normalized values	―	$S_{B}\left(\%\right)$	$S_{W}\left(\%\right)$	$S_{N}\left(\%\right)$	$S_{F}\left(\%\right)$

**Table 7:** Insert condition classification results. (cutting speed 318 m/min, depth of cut 1.8 mm, cutting feed 0.19 mm/rev).

	Weight Values	Built-Up Edge	Flank Wear	Normal	Fracture
Model 1	1.36 × 10^−8^	1	0	0	0
Model 2	1.55 × 10^−6^	0	1	0	0
Model 3	3.39 × 10^−5^	0	1	0	0
Model 4	7.53 × 10^−8^	0	1	0	0
Model 5	5.00 × 10^−4^	1	0	0	0
Model 6	1.34 × 10^−5^	1	0	0	0
Model 7	5.97 × 10^−5^	1	0	0	0
Model 8	9.10 × 10^−3^	1	0	0	0
Model 9	9.90 × 10^−1^	1	0	0	0
Weighted sum values	―	0.9997	3.55 × 10^−5^	0	0
Normalized values	―	99.9964%	0.0036%	0	0
Classification result	Built-up edge				

**Table 8:** Insert condition classification results. (cutting speed 290 m/min, depth of cut 1.9 mm, cutting feed 0.23 mm/rev).

Actual Class	Number of Signals	Insert Condition Classification			
		Built-Up Edge	Flank Wear	Normal	Fracture
Built-up edge	6	5	-	-	1
Flank wear	6	-	6	-	-
Normal	6	-	2	4	-
Fracture	6	-	1	-	5

**Table 9:** Insert condition classification results. (cutting speed 323 m/min, depth of cut 2.3 mm, cutting feed 0.14 mm/rev).

Actual Class	Number of Signals	Insert Condition Classification			
		Built-Up Edge	Flank Wear	Normal	Fracture
Built-up edge	4	3	-	-	1
Flank wear	4	-	2	-	2
Normal	4	-	-	3	1
Fracture	3	3	-	-	-

**Table 10:** Insert condition classification results. (cutting speed 306 m/min, depth of cut 1.1 mm, cutting feed 0.22 mm/rev).

Actual Class	Number of Signals	Insert Condition Classification			
		Built-Up Edge	Flank Wear	Normal	Fracture
Built-up edge	4	4	-	-	-
Flank wear	3	-	-	3	-
Normal	3	-	1	2	-
Fracture	4	2	-	1	1

**Table 11:** Experimental results comparison table.

Cutting Speed (m/min)	Depth of Cut (mm)	Cutting Feed (mm/rev)	Inside/Outside of Experimental Ranges	Cutting Force (N)	Classification Rate (%)
285	1.1	0.17	Inside	225	50.00%
318	1.8	0.19	Inside	399	92.86%
285	1.6	0.18	Inside	341	81.25%
290	1.9	0.23	Inside	484	83.30%
323	2.3	0.14	Outside	409	53.30%
305	1.4	0.23	Inside	357	87.50%
306	1.1	0.22	Inside	271	50.00%
310	1.2	0.17	Inside	245	42.00%

**Table 12:** Classification results of comparative experiments.

Cutting Speed (m/min)	Depth of Cut (mm)	Cutting Feed (mm/rev)	Cutting Force (N)	Classification Rate System 1	Classification Rate System 2
285	1.1	0.17	225	50.00%	35.71%
318	1.8	0.19	399	92.86%	21.43%
285	1.6	0.18	341	81.25%	18.75%
290	1.9	0.23	484	83.30%	25.00%
323	2.3	0.14	409	53.30%	26.32%
305	1.4	0.23	357	87.50%	26.67%
306	1.1	0.22	271	50.00%	31.25%
310	1.2	0.17	245	42.00%	28.57%

## Nomenclature

a:	Depth of cut (mm)
ACC:	Resultant PSD distribution
$ACC_{x}$:	PSD distribution of x-axis accelerometer signal
$ACC_{y}$:	PSD distribution of y-axis accelerometer signal
$ACC_{z}$:	PSD distribution of z-axis accelerometer signal
BPNN:	Backpropagation neural network
C:	Covariance matrix of the training set
F:	Calculated cutting force (N)
f:	Cutting feed (mm/rev)
$\gamma_{0}$:	Rake angle
$h_{m}$:	Chip thickness (mm)
$k_{c}$:	Specific cutting force (N/mm^2^)
$k_{c1}$:	Specific cutting force (N/mm^2^)
$K_{r}$:	Edge angle
$\lambda_{i}$:	Eigenvalue
$m_{c}$:	Non-dimensional factor
PCA:	Principal component analysis
$PI_{i}$:	Weight value
PSD:	Power spectral density
q:	Ratio of the total variance
S:	Training set
$S_{ij}$:	Insert condition classification
$S_{B}$:	Weighted sum value for the built-up edge insert condition
$S_{F}$:	Weighted sum values for the fracture insert condition
$S_{N}$:	Weighted sum value for the normal insert condition
$S_{W}$:	Weighted sum value for the flank wear insert condition
u:	Unitless coefficient
$v_{i}$:	Eigenvector
W:	Transformation matrix
x:	Test data
$x_{i}$:	Training data
$\bar{X}$:	Average of the training set
y:	Transformed data

## Appendix A

**Table A1:** List of devices in the experimental setup.

Device	Model
CNC Lathe	YCM GT-200MA
Accelerometer	PCB Piezotronics 356A15
Data acquisition	NI-9234
Thermometer	HILA TN-49SCG
Toolholder	Sandvik PCLNR 2525M 12
Inserts	CNMG 120408 (produced by Mitsubishi Materials, Tungaloy, Kennametal, and Kyocera Precision Tools)
Laptop computer	ASUS GL502VS

## Appendix B

**Table A2:** Composition of the Al6061 material used in this study (unit: %).

Si	Fe	Cu	Mn	Mg	Cr	Zn	Ti	Al
0.4–0.8	0.7	0.15–0.4	0.15	0.8–1.2	0.04–0.35	0.25	0.15	Others
